# Supplementary material for: A Recurrent Inflammatory Myofibroblastic Tumor-like Lesion of the Splenic Capsule in a Kitten: Clinical, Microscopic and Ultrastructural Description
Source: Vet Sci. 2021 Nov 12;8(11):275. doi: 10.3390/vetsci8110275 (PMC8621069; doi:10.3390/vetsci8110275)
Supplement: Supplementary file 1 [file vetsci-08-00275-s001.zip › vetsci-1405552-supplementary.pdf]

veterinary sciences

MDPI

Case report

A Recurrent Inflammatory Myofibroblastic Tumor-like Lesion of the Splenic Capsule in a Kitten: Clinical, Microscopic and Ultrastructural Description

Silvia Ferro, David Chiavegato, Piergiorgio Fiorentin, Valentina Zappulli and Stefano Di Palma

Supplementary Materials

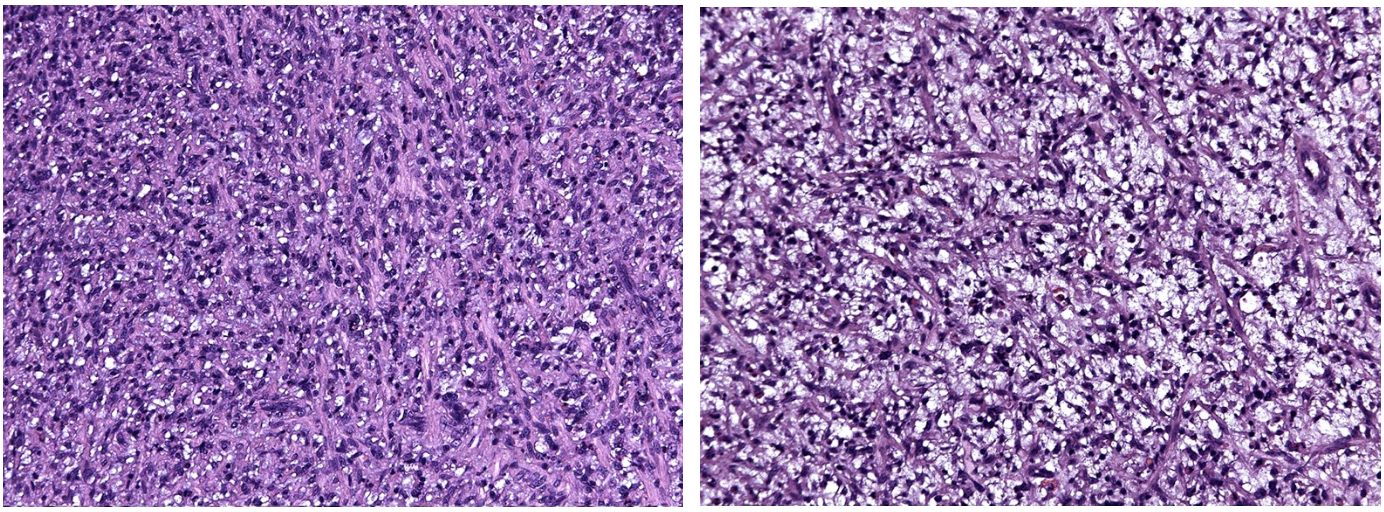

(**a**) (**b**)

Figure S1. Inflammatory myofibroblastic tumor, cat. Spindle cells are embedded in a variably compact (**a**) and loose (**b**) matrix. Hematoxylin Eosin.

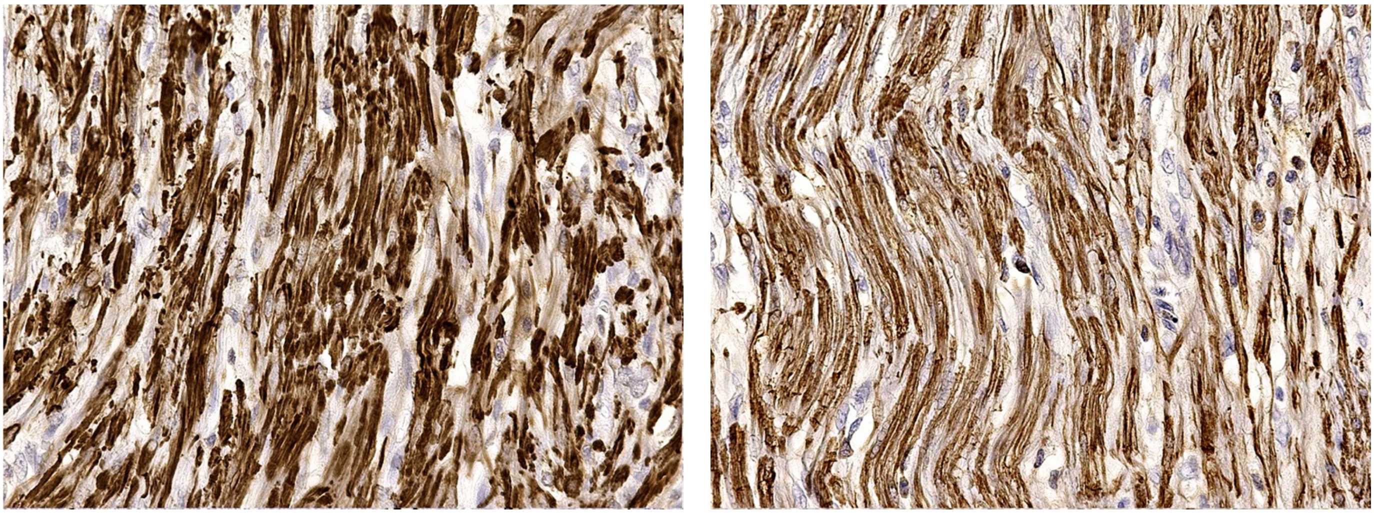

(**a**) (**b**)

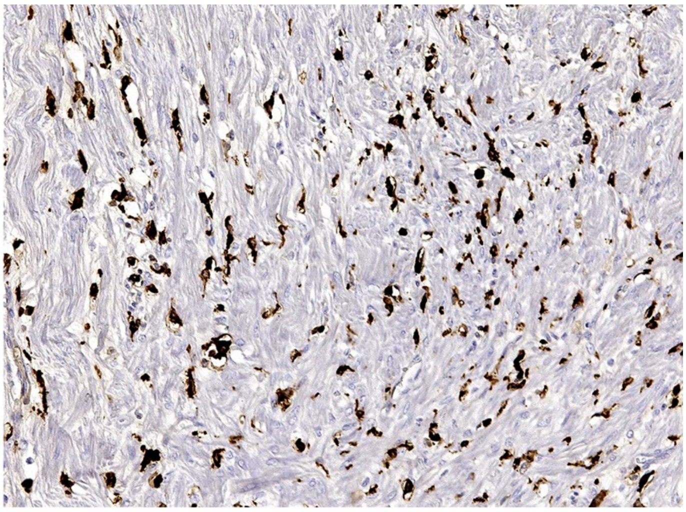

(**c**)

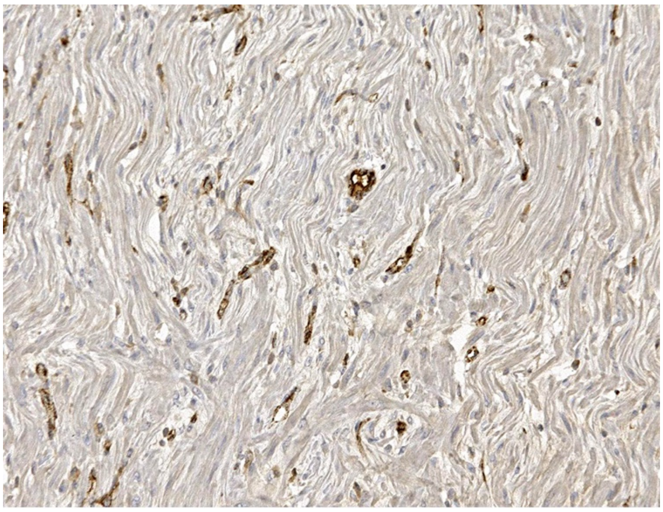

(**d**)

Figure S2. Inflammatory myofibroblastic tumor, cat. Immunohistochemistry (IHC). (**a**) Atypical cells diffusely show strong cytoplasmic immunolabeling for desmin. (**b**) Neoplastic cells also show moderate cytoplasmic immunolabeling for calponin. (**c**) Scattered among the spindle population there are histiocytes with marked cytoplasmic immunolabeling for HLA-dr. (**d**) Immunohistochemistry reveals frequent disseminated capillaries. IHC for VWF.

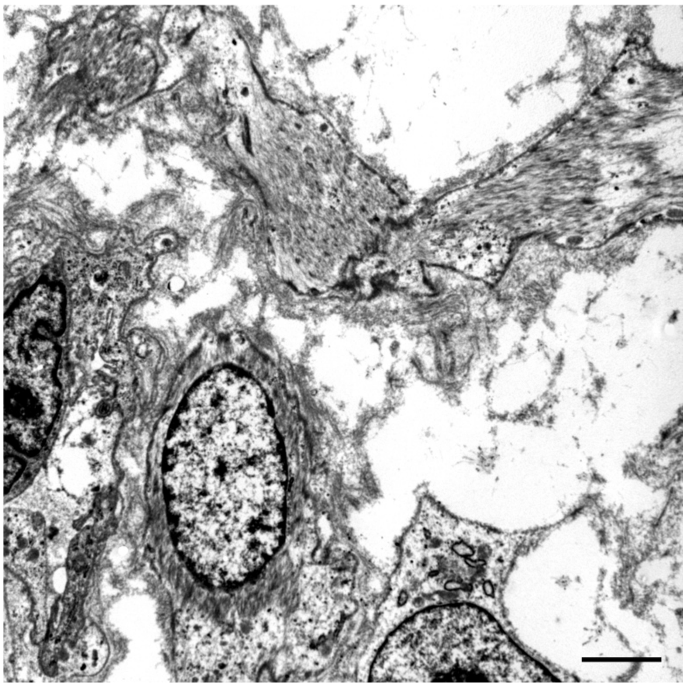

(**a**)

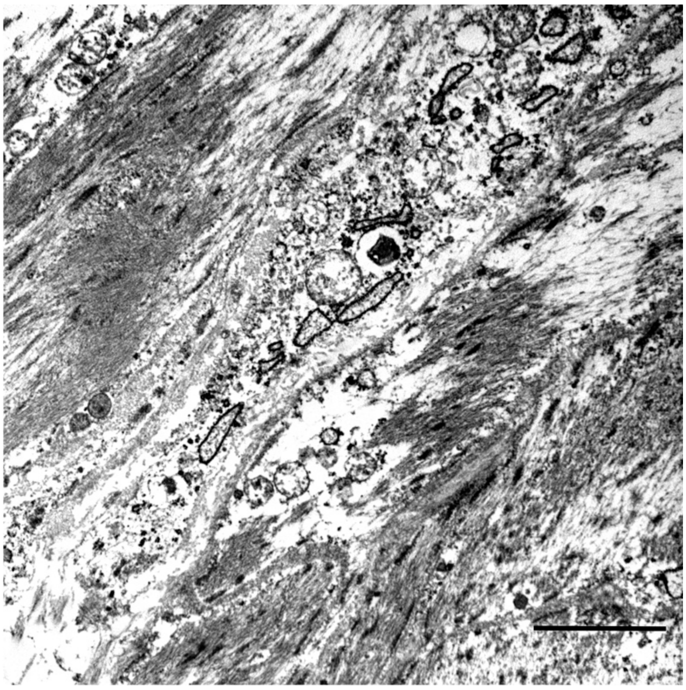

(**b**)

Figure S3. Inflammatory myofibroblastic tumor, cat. Transmission Electron Microscopy. (**a**) A myofibroblastic spindle cell (arrow) with fibrillar cytoplasm embedded in an edematous and fibrillar matrix. Bar = 2 microns. (**b**) Densely fibrillar cytoplasm of the spindle population. Bar = 2 microns.
